# Interaction of uromodulin and complement factor H enhances C3b inactivation

**DOI:** 10.1111/jcmm.12872

**Published:** 2016-04-26

**Authors:** Maojing Liu, Yaqin Wang, Fengmei Wang, Min Xia, Ying Liu, Yuqing Chen, Ming‐Hui Zhao

**Affiliations:** ^1^ Renal DivisionDepartment of MedicinePeking University First HospitalBeijingChina; ^2^ Institute of NephrologyPeking UniversityBeijingChina; ^3^ Key Laboratory of Renal DiseaseMinistry of Health of ChinaBeijingChina; ^4^ Key Laboratory of Chronic Kidney Disease Prevention and TreatmentMinistry of EducationBeijingChina; ^5^ Peking‐Tsinghua Center for Life SciencesBeijingChina

**Keywords:** uromodulin, Tamm‐Horsfall protein, complement factor H, chronic kidney disease

## Abstract

Recent studies suggest that uromodulin plays an important role in chronic kidney diseases. It can interact with several complement components, various cytokines and immune system cells. Complement factor H (CFH), as a regulator of the complement alternative pathway, is also associated with various renal diseases. Thus, we have been suggested that uromodulin regulates complement activation by interacting with CFH during tubulointerstitial injury. We detected co‐localization of uromodulin and CFH in the renal tubules by using immunofluorescence. Next, we confirmed the binding of uromodulin with CFH *in vitro* and found that the affinity constant (K_D_) of uromodulin binding to CFH was 4.07 × 10^−6^M based on surface plasmon resonance results. The binding sites on CFH were defined as the short consensus repeat (SCR) units SCR1–4, SCR7 and SCR19–20. The uromodulin‐CFH interaction enhanced the cofactor activity of CFH for factor I‐mediated cleavage of C3b to iC3b. These results indicate that uromodulin plays a role *via* binding and enhancing the function of CFH.

## Introduction

Uromodulin (UMOD or Tamm‐Horsfall protein), is exclusively expressed in the epithelial cells of the thick ascending limb and early distal convoluted tubule of the kidney 1. Recent studies suggested that uromodulin plays an important role in renal diseases, such as UMOD gene mutation associated kidney disease 2, acute kidney injury 3, 4, and chronic kidney disease 5, 6, 7, 8, 9, 10, 11. A patient with a UMOD gene mutation associated kidney disease exhibited tubular atrophy and interstitial fibrosis 2. The kidney in THP ablation mice also showed additional necrotic tubular cell death and interstitial neutrophil infiltration compared to wild‐type mice after ischaemia‐reperfusion injury 4. Furthermore, a lower urinary uromodulin level has been associated with an increased risk of rapid eGFR decline and more severe tubulointerstitial changes in IgA nephropathy 11. These findings indicate a protective role of uromodulin against tubulointerstitial injury, in which several pathways are involved. Among these potential mechanisms, uromodulin has been gradually recognized as an immune regulatory protein because of its ability to bind inflammatory cytokines, complement 1q 12, 13, 14, and interact with neutrophils, monocytes, lymphocytes, and myeloid dendritic cells 15, 16, 17. Complement activation is involved in tubulointerstitial injury 18; complement factor H (CFH), a crucial factor regulating the alternative complement pathway 19, may bind to tubular epithelial cells and inhibit complement activation in ischaemic renal injury 20.Thus, we have been suggested that uromodulin regulates complement activation by interacting with CFH during tubulointerstitial injury. We first detected the co‐localization of uromodulin and CFH in the kidney, then explored the binding of uromodulin to CFH, and finally investigated the influence of the uromodulin–CFH interaction on cofactor activity of CFH in the cleavage of C3b by factor I.

## Materials and methods

### Reagents

The commercial CFH, factor I, and C3b were purchased from Merck (Kenilworth, NJ, USA). Factor H short consensus repeat (SCR) units (SCR1–4, SCR7, SCR11‐14, and SCR19–20) were produced by Gene‐Script Corporation (Piscataway, Nanjing, China). Antibodies used in this study included a goat anti‐human CFH polyclonal antibody (Merck), a mouse anti‐human CFH monoclonal antibody (US Biological, Salem, MA, USA), a rabbit anti‐human uromodulin polyclonal antibody (Biomedical Technologies Inc., Stoughton, MA, USA), a mouse anti‐human uromodulin monoclonal antibody (Cedarlane, Burlington, NC, USA), and species appropriate secondary antibodies (anti‐mouse IgG‐alkaline phosphatase conjugated antibodies and anti‐rabbit IgG‐alkaline phosphatase conjugated antibodies) from Sigma‐Aldrich (St. Louis, MO, USA). TRITC‐conjugated rabbit anti‐goat IgG (Zhongshan Biotech, Guangdong, China) and FITC‐conjugated rabbit anti‐mouse IgG (Abcam, Cambridge, UK) were also used when necessary.

### Purification of uromodulin from human urine

Uromodulin was purified from the urine of healthy volunteers (two males and two non‐pregnant females) according to a previous report 21. The purified uromodulin was lyophilized and stored at −80°C. The purity and relative molecular weight of the uromodulin were confirmed by 10% SDS‐PAGE with Coomassie blue staining and Western blotting.

### Immunofluorescence

Immunofluorescence staining of uromodulin and CFH was performed on paraffin‐fixed sections from renal biopsy tissues of IgA nephropathy patients. The paraffin section was deparaffinized using xylene and dehydrated with ethanol. Next, the antigen was retrieved by incubation with pepsin. After blocking with PBS containing 3% bovine serum albumin for 30 min, the sections were incubated overnight at 4°C with primary goat anti‐human CFH antibody (1:100, diluted in PBS) and mouse anti‐human uromodulin antibody (1:100, diluted in PBS). Next, the sections were washed three times for 10 min in PBS. Bound antibodies were detected with TRITC–conjugated rabbit anti‐goat IgG (1:50, for FH staining) and FITC–conjugated rabbit anti‐mouse IgG (1:100, for uromodulin staining). Nuclei were stained with DAPI. All the sections were viewed using a laser scanning confocal microscope (Zeiss LSM780; Heidenheim, Germany).

### Binding of uromodulin with CFH and CFH SCRs

Binding of uromodulin to CFH and CFH SCRs were analysed by microtiter plate binding assays. Briefly, 96‐well microtiter plates (Nalge‐Nunc, Rochester, NY, USA) were coated with CFH (4 μg/ml) or different CFH SCRs (4 μg/ml) in 0.1 M carbonate buffer (pH 9.6) overnight at 4°C. Control wells were incubated with buffer alone. The reaction volume was 100 μl and plates were washed three times with 0.01 M PBS containing 0.1% Tween 20. Various concentrations of uromodulin were added, and then incubated at 37°C for 1 h. Rabbit anti‐human uromodulin polyclonal antibody was added to detect the bound uromodulin. Finally, appropriate secondary anti‐species AP‐conjugated Abs was added, and the plates were incubated at 37°C for 1 h. The colour of the reaction was read at an OD of 405 nm.

An inhibition assay was conducted to test the ability of soluble CFH to compete with fixed‐CFH–uromodulin binding. Soluble CFH (from 0 to 2 μg/ml) was incubated with uromodulin (10 μg/ml) at 37°C for 1 h before loading onto CFH‐coated wells.

### Binding of uromodulin to immobilized CFH with real‐time surface plasmon resonance (SPR) spectroscopy

Surface plasmon resonance was carried out on a Biacore 3000 instrument and data were evaluated with BIA evaluation 4.1 software (Biacore AB, Uppsala, Sweden). The steady‐state affinity constant (K_D_) was calculated using BIA evaluation 4.1 software. Factor H was diluted to 10 μg/ml in 10 mM sodium acetate (pH 5.0) and immobilized on CM5 chip surfaces (~600 RU) using the amine coupling method. The fluid state buffer was HBS‐EP (pH 7.4, 0.01 M HEPES, 0.15 M NaCl, 3 mM ethylenediaminetetraacetic acid, and 0.05% Surfactant P‐20). Intact human uromodulin was diluted with ultrapure water and injected at a flow rate of 30 μl/min. The solution buffer was used as a blank control. No regeneration was required.

### Influence of uromodulin on CFH‐mediated C3b inactivation

We tested whether uromodulin affected CFH–C3b binding. CFH was incubated with or without uromodulin in the buffer at 37°C for 1 h, and then the mixture was added to C3b‐coated wells. After 1 h of incubation at 37°C, bound CFH was detected with goat anti‐human CFH antibody, followed by appropriate secondary anti‐species AP‐conjugated Abs. After incubating the reaction for 1 h at 37°C, absorbance was measured at OD 405 nm.

The CFH‐mediated C3b inactivation assay, including CFH (1 μg), factor I (50 ng), C3b (3 μg) and uromodulin (0, 4, 8 and 16 μg), was conducted in a final volume of 20 μl and incubated at 37°C for 30 min. Experiments conducted in the absence of CFH were performed as negative controls. After incubation, the samples were heated for 5 min at 95°C in reducing buffer containing β‐mercaptoethanol and run on a 10% SDS‐PAGE gel. C3b and its cleavage products were detected by western blotting using a polyclonal anti‐C3c antibody (Dako Cytomation, Carpinteria, CA, USA).

### Statistical analyses

Descriptive statistics are presented as the mean values ± SD. One‐way anova (multiple groups) was used for comparison. All data were analysed using SPSS 13.0 statistical software (SPSS, Inc., Chicago, IL, USA).

## Results and discussion

### Co‐localization of uromodulin and CFH in renal tubular cells

**Figure 1:**
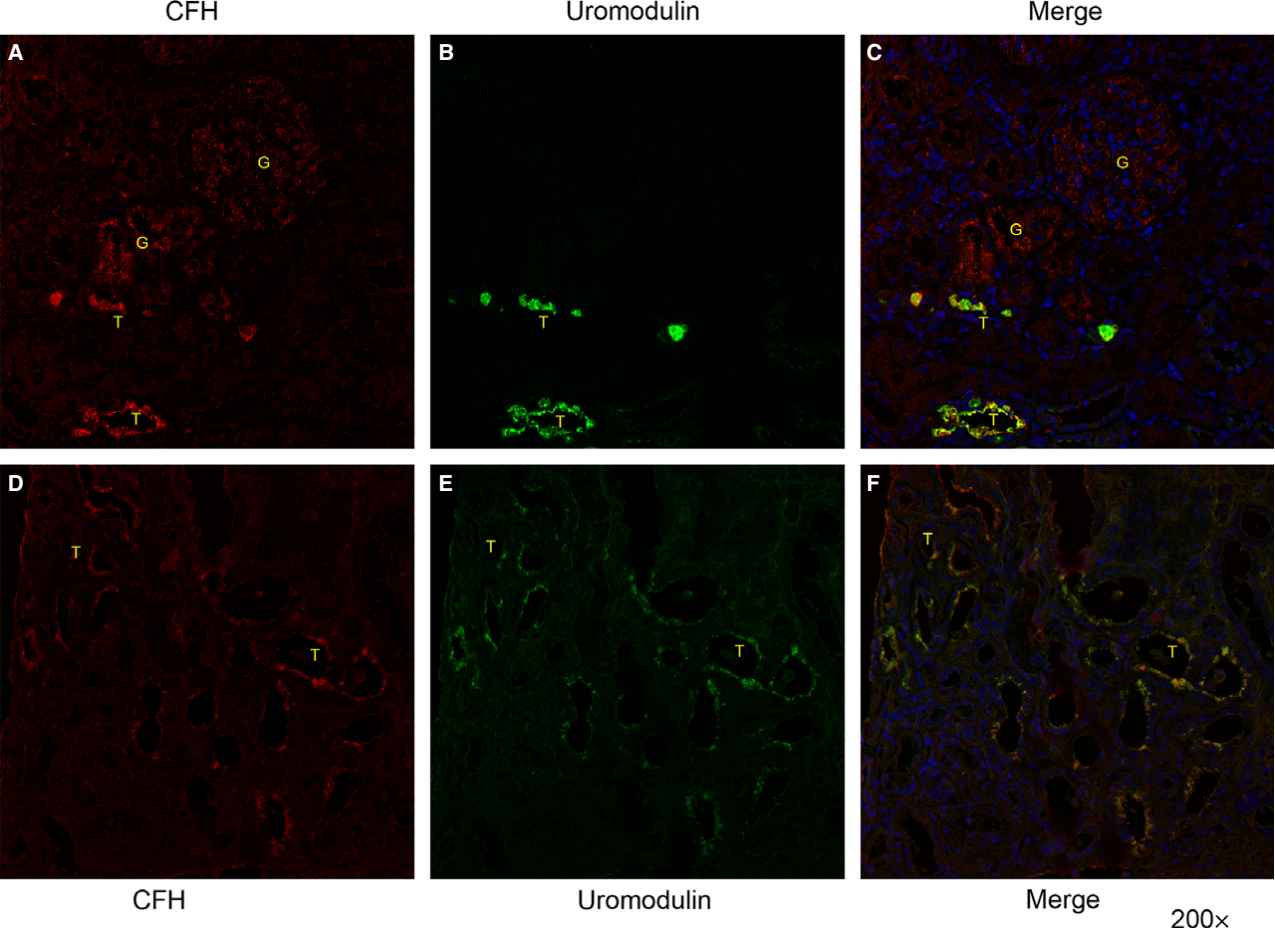
Co‐localization of uromodulin and complement factor H on renal tubules of IgA nephropathy. Paraffin kidney sections from renal biopsy tissue of IgA nephropathy patients are shown. Representative field is from the cortex, with glomeruli (G) and tubules (T). (**A** and **D**) Complement factor H with immunofluorescence staining (red) is distributed on the glomeruli and tubules. (**B** and **E**) Uromodulin with immunofluorescence staining (green) is distributed on the tubules only. (**C** and **F**) Double‐labelled uromodulin and complement factor H yielded a yellow signal. Nucleus was stained with DAPI (blue). (**A**–**C)** From an IgAN patient without tubular atrophy and interstitial fibrosis. (**D**–**F)** From an IgAN patient with focal tubular atrophy and interstitial fibrosis.

We detected CFH in the glomeruli, tubules and interstitium (Fig. 1A and D, Figure S1), and uromodulin in the tubules only (Fig. 1B and E). Both CFH and uromodulin were mainly distributed on the apical membrane of the renal tubules. We also found that uromodulin (green colour) and CFH (red colour) co‐localized in the same tubular cells and generated a yellow colour (Fig. 1C and F). Figure 1A–C are images from an IgAN patient without obvious renal tubular atrophy and interstitial fibrosis. Figure 1D–F are images from another IgAN patient with obvious renal tubular atrophy and interstitial fibrosis. Figure S2A–C show the early stage of diabetic nephropathy (DN), only showing thick glomerular basement membrane. Figure S2D–F shows the later stage of DN, with glomerular sclerosis and tubulointerstitial fibrosis. More CFH and uromodulin as well as co‐localization were detected in patients with severe pathological changes either in DN or IgAN.

### Binding of uromodulin to CFH

**Figure 2:**
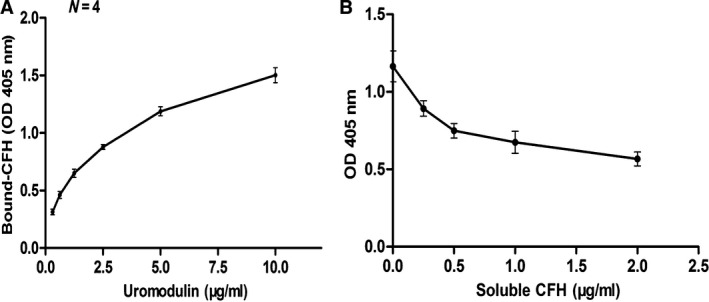
Analysis of UMOD–CFH interaction by microtiter plate binding assays and surface plasmon resonance. Results are presented as the mean values ± SD from three independent experiments performed in duplicate wells. Binding intensity is shown as absorbance readings at 405 nm. (**A**) Dose‐dependent binding of uromodulin to CFH‐coated plates. (**B**) Competitive inhibition assay of UMOD–CFH binding. Soluble complement factor H with different concentrations was first incubated with10 μg/ml uromodulin. Then, the mixture was added to CFH‐coated plates.

**Figure 3:**
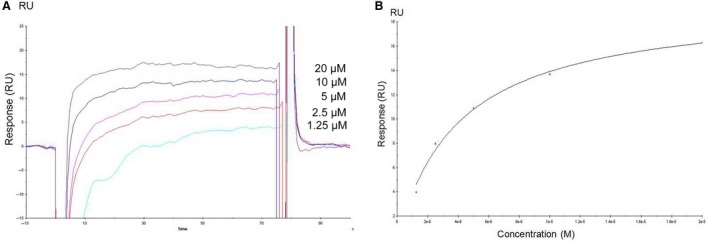
Analysis of uromodulin–CFH interaction by surface plasmon resonance. (**A**) Dose–response analysis of intact uromodulin binding to immobilized CFH by SPR. Sensorgram (response in RU *versus* time in s) obtained for 1.25, 2.5, 5, 10 and 20 μM uromodulin injections across immobilized CFH. (**B**) Steady‐state affinity of uromodulin to immobilized CFH of (**A**). Affinity constant K_D_ = 4.07 × 10^−6^M.

We detected the binding of uromodulin to immobilized CFH using microtiter plate binding assays. We found that uromodulin in the liquid phase bound to pre‐fixed CFH in a dose‐dependent manner (Fig. 2A). Various concentrations of CFH were incubated with 10 μg/ml uromodulin at 37°C for 1 h before loading onto CFH‐coated plates. The results showed that CFH in the liquid phase could inhibit the binding of uromodulin to immobilized CFH, and the inhibition increased with increasing soluble CFH concentration. The inhibition assay revealed a specific interaction between CFH and uromodulin (Fig. 2B). SPR was carried out to confirm whether the binding of CFH and uromodulin occurred in a dose‐dependent manner. When the solution buffer consisted of ultrapure water, no regeneration was required. The K_D_ of intact uromodulin binding to immobilized CFH was 4.07 × 10^−6^M. Solution buffer was used as a blank control (Fig. 3A and B).

In the SPR experiment, we found that uromodulin dissolves easily in water but not in HBS‐EP. The results for when the solution buffer was ultrapure water, are shown in Fig. 3A and B. When the solution buffer was HBS‐EP, uromodulin easily formed polymers, and each sample was suspended using an ultrasonic disintegrator (Sonics & Materials, Newtown, CT, USA) for 5 min before injection to deploymerize. Surface regeneration was achieved by injection of 30 μl of 10 mM NaOH, giving K_D_ of 1.33 × 10^−10^ M. The structure of the uromodulin maybe changed under ultrosonic conditions, likely affecting the interaction between CFH and uromodulin.

### Mapping of uromodulin–CFH interaction sites

**Figure 4:**
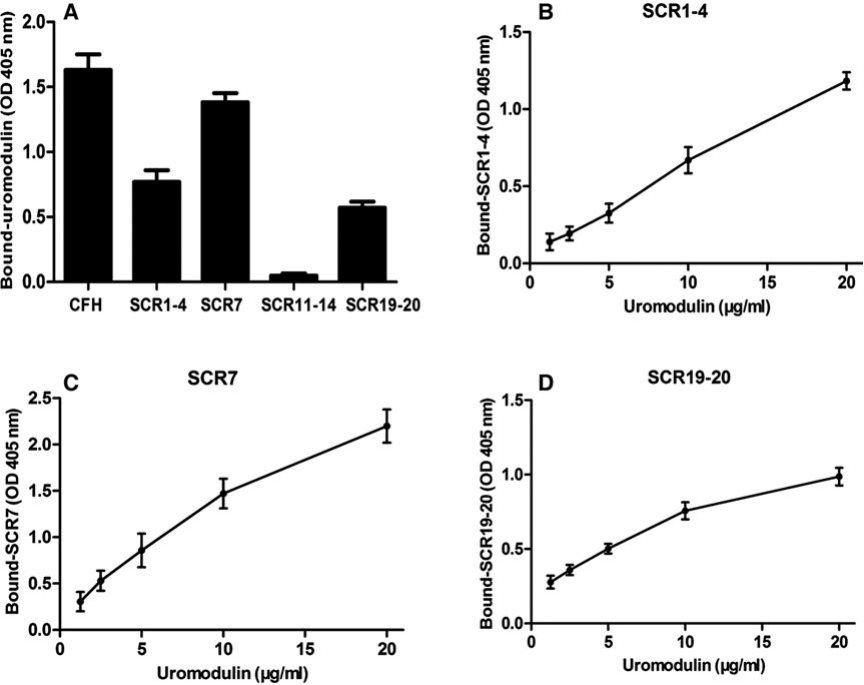
Mapping of UMOD–CFH interaction sites on CFH. Results are presented as the mean values ± SD from at least three independent experiments performed in duplicate wells. (**A**) Uromodulin (10 μg/ml) was incubated with full‐length or short consensus repeats of CFH (4 μg/ml). (**B**) Dose‐dependent binding of uromodulin to CFH SCR1–4 coated wells. (**C**) Dose‐dependent binding of uromodulin to CFH SCR7 coated wells. (**D**) Dose‐dependent binding of uromodulin to CFH SCR19‐20 coated wells.

Next, we tested which region of CFH could bind to uromodulin. We used four typical CFH SCRs (SCR1‐4, SCR7, SCR11‐14 and SCR19‐20) to repeat the binding assay with uromodulin. Three of the four SCRs, SCR1‐4, SCR 7 and SCR 19‐20 bound to uromodulin (Fig. 4A) in a dose‐dependent manner (Fig. 4B).

### Influence of uromodulin–CFH interaction on C3b inactivation

It is well‐known that CFH binds with C3b and accelerate C3b inactivation as a cofactor of factor I. To identify whether the uromodulin–CFH interaction influences the cofactor activity of CFH in C3b inactivation, we first explored whether CFH–C3b binding could be altered in the presence of uromodulin, and then investigated the influence of uromodulin on C3b degeneration.

**Figure 5:**
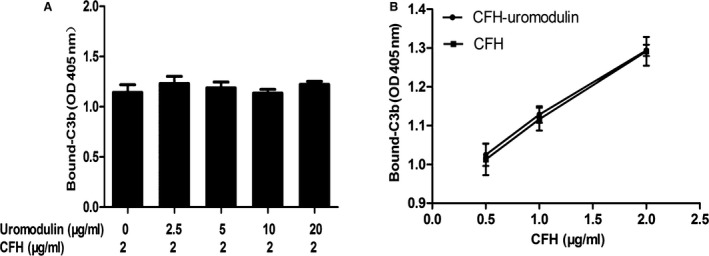
Influence of uromodulin on CFH–C3b interaction. Results are presented as the mean values ± SD from three independent experiments in duplicate wells. Normalized data were compared by one‐way anova analysis. (**A**) CFH (2 μg/ml) was first incubated with uromodulin (0, 2.5, 5, 10, 20 μg/ml). Next, the mixture was added to C3b‐coated plates. (**B**) Dose‐dependent binding of CFH–C3b, with or without uromodulin (10 μg/ml).

We incubated CFH and uromodulin together in C3b‐coated wells to explore whether CFH–C3b binding was affected by the presence of uromodulin. We first used a fixed CFH level (2 μg/ml) with varying uromodulin concentrations (2.5, 5, 10, 20 μg/ml), and then used a fixed uromodulin concentration (10 μg/ml) with varying CFH levels. The results showed that CFH binding to C3b was dose‐dependent and that the presence of uromdulin had little effect on their binding (Fig. 5A and B).

**Figure 6:**
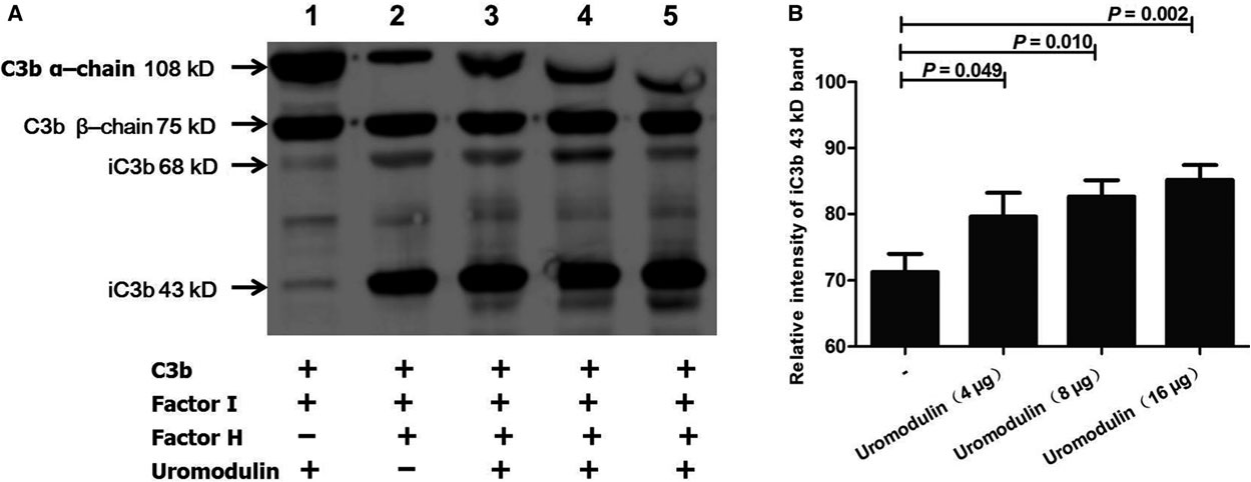
Influence of uromodulin on CFH cofactor activity. The cofactor activity of factor H was assayed in the fluid phase. C3b (3 μg) and factor I (50 ng) was incubated with CFH (1 μg) with different doses of uromodulin (0, 4, 8, 16 μg). (**A**) Western blotting of C3b and its fragments. Lane 1 is the negative control without CFH to test whether there is intrinsic cofactor activity of uromodulin. Lane 2 is the positive control without uromodulin. Lanes 3 to lane 5 show the influence of uromodulin on C3b cleavage. (**B**) Densitometric analyses of the iC3b 43 kD band. Results are presented as the mean values ± SD of three independent experiments in duplicate wells. The first black column on the left represents the control without uromodulin. The intensity of the iC3b 43 kD band clearly increased with uromodulin dose (*P* < 0.05).

Next, we explored whether the uromodulin–CFH interaction influences the cofactor activity of CFH upon C3b inactivation. C3b typically contains two fragments, an α‐chain (108 kD) and a β‐chain (75 kD). C3b becomes inactivated when it is cleaved into two fragments weighing 68 kD and 43 kD by the cofactor action of CFH and factor I. Thus, we added uromodulin to the CFH‐factor I‐C3b system, and then detected the 68 kD and 43 kD fragments of C3b *via* western blotting. As shown in Fig. 6A, C3b was cleaved to the 68 kD and 43 kD fragments when it was incubated with CFH and factor I. Uromodulin enhanced this action, and more 43 kD fragments were generated than in the absence of uromodulin; this action increased when increasing uromodulin concentration (*P* < 0.05, Fig. 6A and B). However, uromodulin had no effect on C3b inactivation without CFH (Fig. 6A).

In this study, we observed a direct interaction between uromodulin and CFH. The two molecules co‐localized in human tubular cells and a direct interaction was confirmed using an *in vitro* assay. We also observed the direct binding of uromodulin to CFH SCR1‐4, SCR7, and SCR19–20. Although uromodulin did not influence the binding of CFH to C3b, it enhanced the cofactor activity of CFH in the cleavage of C3b by factor I.

Complement factor H was mainly detected in the blood vessels of the renal cortex and mesangial cells in the adult kidneys 22. However, CFH also showed an obvious tubular distribution in the fetal kidneys 22. CFH has been recognized as an important factor in tubulointerstitial injury because of its ability to bind with tubular epithelial cells and inhibit excessive complement activation 20, 23. It may originate in the blood, filtered into the lumen and interstitium, and taken up by tubular cells. However, reports from other researchers demonstrated that CFH can be generated by mesangial cells, epithelial cells and proximal tubular cells 24, 25, 26. Although renal tubular cells expressed various complement components and complement receptors, such as C2, C3, C4, factor B, factor H, CR1, CR3 and CD88 25, 27, the exact mechanism of CFH binding with renal tubular cells are unclear. We detected CFH in the glomeruli, tubules and interstitium in this study. Co‐localization of CFH with uromodulin in our results demonstrated that CFH was distributed in the thick ascending limb of the kidney. We also found more CFH, uromodulin and co‐localization of these two proteins in patients with severe pathological change either in DN or IgAN. Complement factor H, as a crucial factor inhibiting complement activation, targets the complement activation both in the fluid phase and on the cell surface. CFH will aggregate in the area with more complement activation to protect the cells against injury because of overwhelmed complement activation. Thus there will be different results, recovery from injuries or resulting in pathological changes. In our study, we only used the renal biopsy tissue to do the immunofluorescence studies, the increased expression of tubular CFH and uromodulin in association with severe injury is more probably related with disease severity but not prognosis. However, the increased expression of CFH and uromodulin indicated both proteins involved in tubulointerstitial injury.

Uromodulin is mainly expressed on the cell surface of the thick ascending limb and its binding with CFH indicates that CFH attaches to the cell surface. We further found that uromodulin bound CFH at three major sites, including SCR1–4, SCR7 and SCR19–20. CFH SCR7 was previously reported as a major binding site of CRP 19, 28. SCR1–4 of CFH was not only the binding site for C3b but also a functional domain for the cofactor activity of CFH 29. SCR19–20 mainly bound to heparin, sialic acid and microbial virulence factors in favour of SCR1–4 inhibiting complement activation at the surface of host cells, and thus protecting self‐cells from damage 30. It appeared that the three binding sites were from the N‐terminal to C terminal across the whole CFH protein, and may form a relatively tight combination with uromodulin. All three sites also bind to other factors, and thus may form a dendritic structure covering the cell surface. More importantly, SCR1‐4 of CFH can regulate the function of CFH when bound to C3b. Our study revealed that the presence of uromodulin in the liquid phase did not influence the binding of CFH to C3b, but enhanced CFH‐mediated complement inactivation. It is reasonable to conclude that, after binding to uromodulin, CFH inhibits excessive activation of the alternative complement pathway in the uromodulin‐enriched environment, and thus playing a role in the kidney injury 19. The important role of complement activation in tubulointerstitial injury has been demonstrated during recent years 31. We detected CFH in the glomeruli, tubules and interstitium in this study. It is not fully understood where the CFH comes from. It may originate in the blood, filtered into the lumen and interstitium, or generated by tubular cells. Although the co‐localization of CFH‐UMOD is mainly on apical membrane of tubules, uromodulin is also distributed in the plasma and basolateral membrane 32. The physiological role of each segment of renal tubules has not been fully understood, the interaction and co‐localization of CFH‐UMOD indicate some potential mechanisms in the regulation of tubular injury. Thus, further investigation is needed.

Since it was first discovered to inhibit viral haemagglutination 33, 34, uromodulin has been found to have multiple immune regulatory functions including interactions with neutrophils, monocytes, lymphocytes and myeloid dendritic cells 15, 16, 17, binding to cytokines and complements, such as IL‐1, IL‐2, IL‐8, TNF‐α, C1, C1q and C3 12, 13, 14. In this study, we confirmed that uromodulin bound to immobilized CFH and influenced its function, adding another important factor in complement systems to the family of uromodulin binding proteins.

Renal tubular cells were gradually accepted as important trouble sensors and possibly trouble‐makers in the danger model and involved in epithelium to epithelium tubular cross‐talk 35. Thus, many protein‐recognized receptors or molecules in tubular cells were found to be involved in the injury process. Uromodulin is likely such a molecule in the thick ascending limb.

## Conclusion

In summary, we demonstrated that uromodulin bound specifically with CFH at three sites (SCR1‐4, SCR7, SCR19–20), and this binding enhanced the cofactor activity of CFH during the cleavage of liquid phase C3b by factor I. This finding indicates that uromodulin acts as an important protein that recognizes molecules to regulate innate immunity in the thick ascending limb cells.

## Disclosure

All the authors declared no competing interests.

## Supporting information


**Figure S1** Expression of complement factor H in renal tissue.

**Figure S2** Co‐localization of uromodulin and complement factor H on renal tubules of diabetic nephropathy.

## References

[1] Bachmann S , Koeppen‐Hagemann I , Kriz W . Ultrastructural localization of Tamm‐Horsfall glycoprotein (THP) in rat kidney as revealed by protein A‐gold immunocytochemistry. Histochemistry. 1985; 83: 531–8. 3910623 10.1007/BF00492456

[2] Hart TC , Gorry MC , Hart PS , *et al* Mutations of the UMOD gene are responsible for medullary cystic kidney disease 2 and familial juvenile hyperuricaemic nephropathy. J Med Genet. 2002; 39: 882–92. 12471200 10.1136/jmg.39.12.882 PMC1757206

[3] El‐Achkar TM , McCracken R , Rauchman M , *et al* Tamm‐Horsfall protein‐deficient thick ascending limbs promote injury to neighboring S3 segments in an MIP‐2‐dependent mechanism. Am J Physiol Renal Physiol. 2011; 300: F999–1007. 21228114 10.1152/ajprenal.00621.2010 PMC5504439

[4] El‐Achkar TM , Wu XR , Rauchman M , *et al* Tamm‐Horsfall protein protects the kidney from ischemic injury by decreasing inflammation and altering TLR4 expression. Am J Physiol Renal Physiol. 2008; 295: F534–44. 18495803 10.1152/ajprenal.00083.2008 PMC5504389

[5] Gudbjartsson DF , Holm H , Indridason OS , *et al* Association of variants at UMOD with chronic kidney disease and kidney stones‐role of age and comorbid diseases. PLoS Genet. 2010; 6: e1001039. 20686651 10.1371/journal.pgen.1001039 PMC2912386

[6] Han J , Liu Y , Rao F , *et al* Common genetic variants of the human uromodulin gene regulate transcription and predict plasma uric acid levels. Kidney Int. 2013; 83: 733–40. 23344472 10.1038/ki.2012.449 PMC3687544

[7] Kottgen A , Glazer NL , Dehghan A , *et al* Multiple loci associated with indices of renal function and chronic kidney disease. Nat Genet. 2009; 41: 712–7. 19430482 10.1038/ng.377 PMC3039280

[8] Rasch R , Torffvit O , Bachmann S , *et al* Tamm‐Horsfall glycoprotein in streptozotocin diabetic rats: a study of kidney in situ hybridization, immunohistochemistry, and urinary excretion. Diabetologia. 1995; 38: 525–35. 7489834 10.1007/BF00400720

[9] Thulesen J , Jorgensen PE , Torffvit O , *et al* Urinary excretion of epidermal growth factor and Tamm‐Horsfall protein in three rat models with increased renal excretion of urine. Regul Pept. 1997; 72: 179–86. 9652978 10.1016/s0167-0115(97)01058-6

[10] Tsai CY , Wu TH , Yu CL , *et al* Increased excretions of beta2‐microglobulin, IL‐6, and IL‐8 and decreased excretion of Tamm‐Horsfall glycoprotein in urine of patients with active lupus nephritis. Nephron. 2000; 85: 207–14. 10867535 10.1159/000045663

[11] Zhou J , Chen Y , Liu Y , *et al* Urinary uromodulin excretion predicts progression of chronic kidney disease resulting from IgA nephropathy. PLoS ONE. 2013; 8: e71023. 23990922 10.1371/journal.pone.0071023 PMC3750049

[12] Hession C , Decker JM , Sherblom AP , *et al* Uromodulin (Tamm‐Horsfall glycoprotein): a renal ligand for lymphokines. Science. 1987; 237: 1479–84. 3498215 10.1126/science.3498215

[13] Rhodes DC . Binding of Tamm‐Horsfall protein to complement 1q measured by ELISA and resonant mirror biosensor techniques under various ionic‐strength conditions. Immunol Cell Biol. 2000; 78: 474–82. 11050529 10.1111/j.1440-1711.2000.t01-3-.x

[14] Sherblom AP , Sathyamoorthy N , Decker JM , *et al* IL‐2, a lectin with specificity for high mannose glycopeptides. J Immunol. 1989; 143: 939–44. 2787353

[15] Cavallone D , Malagolini N , Serafini‐Cessi F . Binding of human neutrophils to cell‐surface anchored Tamm‐Horsfall glycoprotein in tubulointerstitial nephritis. Kidney Int. 1999; 55: 1787–99. 10231441 10.1046/j.1523-1755.1999.00439.x

[16] Miyata T , Sugiyama S , Nangaku M , *et al* Apolipoprotein E2/E5 variants in lipoprotein glomerulopathy recurred in transplanted kidney. J Am Soc Nephrol. 1999; 10: 1590–5. 10405216 10.1681/ASN.V1071590

[17] Saemann MD , Weichhart T , Zeyda M , *et al* Tamm‐Horsfall glycoprotein links innate immune cell activation with adaptive immunity *via* a Toll‐like receptor‐4‐dependent mechanism. J Clin Invest. 2005; 115: 468–75. 15650774 10.1172/JCI22720 PMC544039

[18] Hsu SI , Couser WG . Chronic progression of tubulointerstitial damage in proteinuric renal disease is mediated by complement activation: a therapeutic role for complement inhibitors? J Am Soc Nephrol. 2003; 14: S186–91. 12819326 10.1097/01.asn.0000070032.58017.20

[19] Rodriguez de Cordoba S , Esparza‐Gordillo J , Goicoechea de Jorge E , *et al* The human complement factor H: functional roles, genetic variations and disease associations. Mol Immunol. 2004; 41: 355–67. 15163532 10.1016/j.molimm.2004.02.005

[20] Renner B , Ferreira VP , Cortes C , *et al* Binding of factor H to tubular epithelial cells limits interstitial complement activation in ischemic injury. Kidney Int. 2011; 80: 165–73. 21544060 10.1038/ki.2011.115 PMC3133686

[21] Serafini‐Cessi F , Bellabarba G , Malagolini N , Dall'Olio F . Rapid isolation of Tamm‐Horsfall glycoprotein (uromodulin) from human urine. J Immunol Methods. 1989; 120: 185–9. 2500486 10.1016/0022-1759(89)90241-x

[22] Vaziri‐Sani F , Holmberg L , Sjoholm AG , *et al* Phenotypic expression of factor H mutations in patients with atypical hemolytic uremic syndrome. Kidney Int. 2006; 69: 981–8. 16528247 10.1038/sj.ki.5000155

[23] Buelli S , Abbate M , Morigi M , *et al* Protein load impairs factor H binding promoting complement‐dependent dysfunction of proximal tubular cells. Kidney Int. 2009; 75: 1050–9. 19242507 10.1038/ki.2009.8

[24] van den Dobbelsteen ME , Verhasselt V , Kaashoek JG , *et al* Regulation of C3 and factor H synthesis of human glomerular mesangial cells by IL‐1 and interferon‐gamma. Clin Exp Immunol. 1994; 95: 173–80. 8287602 10.1111/j.1365-2249.1994.tb06033.x PMC1534640

[25] Gerritsma JS , Gerritsen AF , De Ley M , *et al* Interferon‐gamma induces biosynthesis of complement components C2, C4 and factor H by human proximal tubular epithelial cells. Cytokine. 1997; 9: 276–83. 9112336 10.1006/cyto.1996.0164

[26] Ren G , Doshi M , Hack BK , *et al* Rat glomerular epithelial cells produce and bear factor H on their surface that is up‐regulated under complement attack. Kidney Int. 2003; 64: 914–22. 12911541 10.1046/j.1523-1755.2003.00188.x

[27] Timmerman JJ , van der Woude FJ , van Gijlswijk‐Janssen DJ , *et al* Differential expression of complement components in human fetal and adult kidneys. Kidney Int. 1996; 49: 730–40. 8648914 10.1038/ki.1996.102

[28] Jarva H , Jokiranta TS , Hellwage J , *et al* Regulation of complement activation by C‐reactive protein: targeting the complement inhibitory activity of factor H by an interaction with short consensus repeat domains 7 and 8‐11. J Immunol. 1999; 163: 3957–62. 10490997

[29] Kuhn S , Zipfel PF . Mapping of the domains required for decay acceleration activity of the human factor H‐like protein 1 and factor H. Eur J Immunol. 1996; 26: 2383–7. 8898949 10.1002/eji.1830261017

[30] Ferreira VP , Pangburn MK , Cortes C . Complement control protein factor H: the good, the bad, and the inadequate. Mol Immunol. 2010; 47: 2187–97. 20580090 10.1016/j.molimm.2010.05.007 PMC2921957

[31] Hato T , Dagher PC . How the innate immune system senses trouble and causes trouble. Clin J Am Soc Nephrol. 2015; 10: 1459–69. 25414319 10.2215/CJN.04680514 PMC4527020

[32] El‐Achkar TM , McCracken R , Liu Y , *et al* Tamm‐Horsfall protein translocates to the basolateral domain of thick ascending limbs, interstitium, and circulation during recovery from acute kidney injury. Am J Physiol Renal Physiol. 2013; 304: F1066–75. 23389456 10.1152/ajprenal.00543.2012 PMC3625838

[33] Tamm I , Horsfall FL Jr . Characterization and separation of an inhibitor of viral hemagglutination present in urine. Proc Soc Exp Biol Med. 1950; 74: 106–8. 15430405

[34] Tamm I , Horsfall FL Jr . A mucoprotein derived from human urine which reacts with influenza, mumps, and Newcastle disease viruses. J Exp Med. 1952; 95: 71–97. 14907962 10.1084/jem.95.1.71 PMC2212053

[35] El‐Achkar TM , Dagher PC . Tubular cross talk in acute kidney injury: a story of sense and sensibility. Am J Physiol Renal Physiol. 2015; 308: F1317–23. 25877507 10.1152/ajprenal.00030.2015 PMC4469890

