# Supplementary figures and images for: Interaction of uromodulin and complement factor H enhances C3b inactivation
Source: J Cell Mol Med. 2016 Apr 26;20(10):1821–8. doi: 10.1111/jcmm.12872 (PMC5020621; doi:10.1111/jcmm.12872)

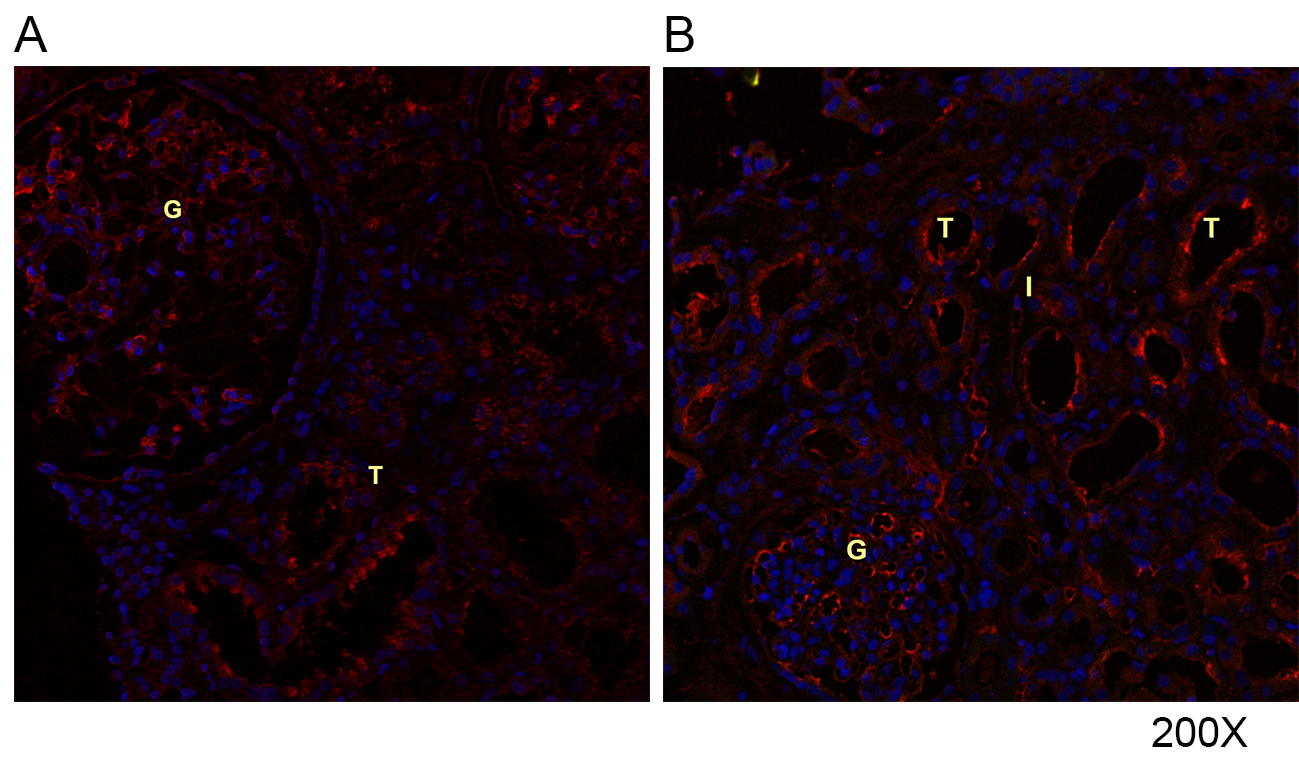

Supplement: Supplementary file 1 — Figure S1 Expression of complement factor H in renal tissue. [file JCMM-20-1821-s001.tif]

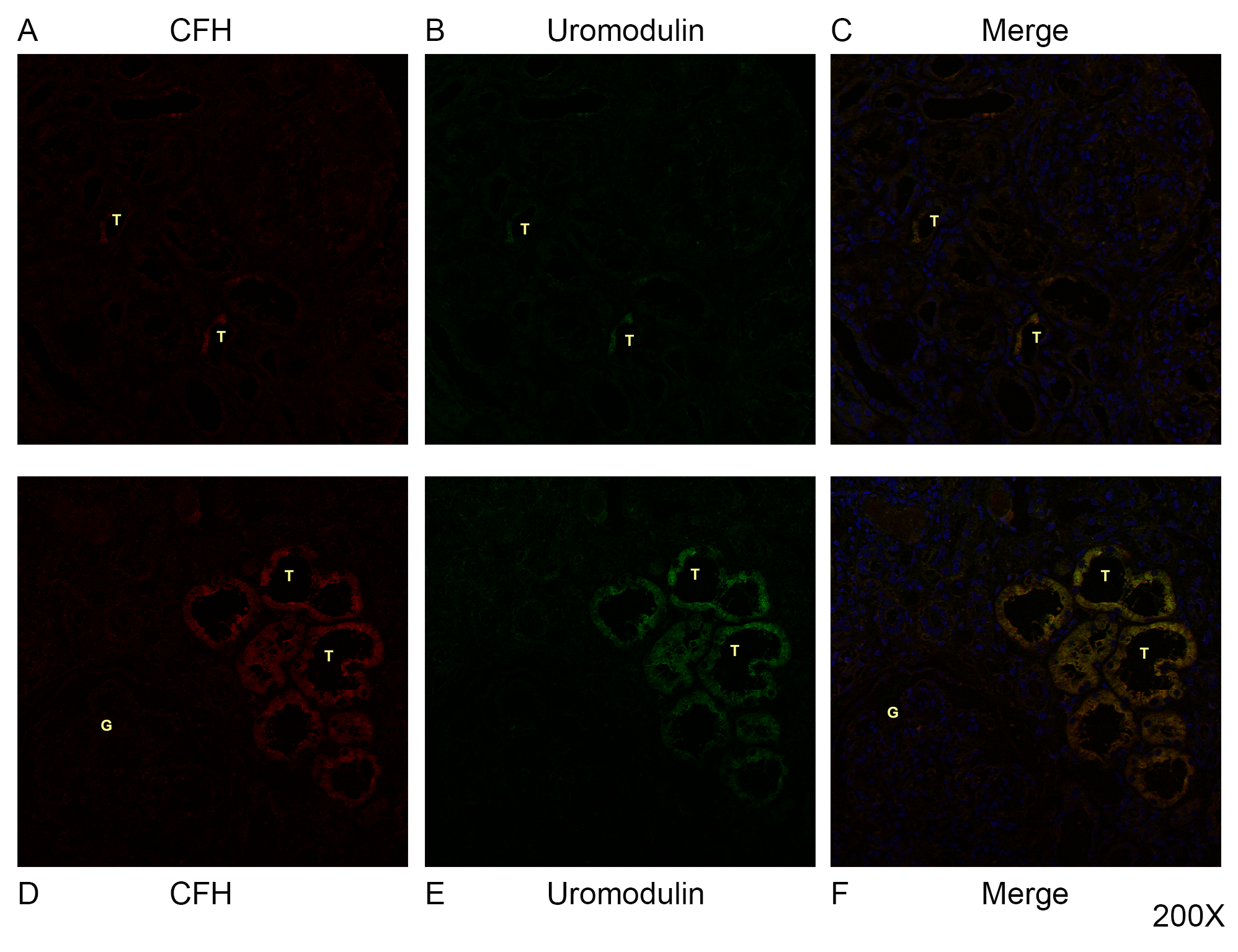

Supplement: Supplementary file 2 — Figure S2 Co‐localization of uromodulin and complement factor H on renal tubules of diabetic nephropathy. [file JCMM-20-1821-s002.tif]
